# Voltage-dependent gating of KCNH potassium channels lacking a covalent link between voltage-sensing and pore domains

**DOI:** 10.1038/ncomms7672

**Published:** 2015-03-30

**Authors:** Éva Lörinczi, Juan Camilo Gómez-Posada, Pilar de la Peña, Adam P. Tomczak, Jorge Fernández-Trillo, Ulrike Leipscher, Walter Stühmer, Francisco Barros, Luis A. Pardo

**Affiliations:** 1 Department of Molecular Biology of Neuronal Signals, Max Planck Institute of Experimental Medicine, Hermann-Rein-Straße 3, 37075 Göttingen, Germany; 2 Departamento de Bioquímica y Biología Molecular, Universidad de Oviedo, Edificio Santiago Gascón, Campus de El Cristo, 33006 Oviedo, Spain; 3 Oncophysiology Group, Max Planck Institute of Experimental Medicine, Hermann-Rein-Straße 3, 37075 Göttingen, Germany; 4 Cluster of Excellence Nanoscale Microscopy and Molecular Physiology of the Brain, 37073 Göttingen, Germany

## Abstract

Voltage-gated channels open paths for ion permeation upon changes in membrane potential, but how voltage changes are coupled to gating is not entirely understood. Two modules can be recognized in voltage-gated potassium channels, one responsible for voltage sensing (transmembrane segments S1 to S4), the other for permeation (S5 and S6). It is generally assumed that the conversion of a conformational change in the voltage sensor into channel gating occurs through the intracellular S4–S5 linker that provides physical continuity between the two regions. Using the pathophysiologically relevant KCNH family, we show that truncated proteins interrupted at, or lacking the S4–S5 linker produce voltage-gated channels in a heterologous model that recapitulate both the voltage-sensing and permeation properties of the complete protein. These observations indicate that voltage sensing by the S4 segment is transduced to the channel gate in the absence of physical continuity between the modules.

## Abstract (web summary)

The pore of voltage-gated ion channels opens in response to membrane depolarization sensed by a separate voltage-sensing domain. Here, Lörinczi *et al*. show that, contrary to assumptions, no physical linker is required to transmit changes from the voltage-sensing to the permeation domain of KCNH channels.

## Graphical abstract

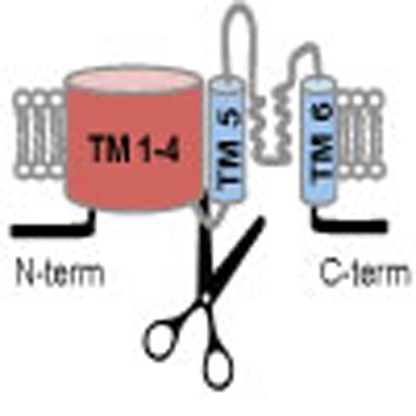

Voltage-gated potassium (K_V_) channels are crucial regulators of cell excitability. They allow potassium to flow along its electrochemical gradient upon depolarization of the plasma membrane. Similar to voltage-gated Na^+^ and Ca^2+^ channels, K_V_ channels have four-fold symmetry that is generated by internal repeats in Na^+^ and Ca^2+^ channels and by independent subunits in K^+^ channels. Thus, K_V_ channels are tetramers, each monomer containing six transmembrane segments (S1 to S6). A modular structure can be recognized in the arrangement of these channels. Functional K_V_ channels are formed by an ensemble of a ‘permeation module’ or pore domain that allows potassium flow, constituted by four S5 and S6 segments and their intervening pore loop, and that is surrounded by a ‘voltage-sensing module’ comprised of four functionally independent voltage-sensor domains^1^,^2^,^3^. The permeation module would be equivalent to the two transmembrane segments of the bacterial KcsA or of the inward rectifier channels, which have little or no intrinsic voltage dependence (reviewed by Vardanyan and Pongs^4^). Voltage dependence is conferred by the rest of the transmembrane protein (segments S1–S4, although intracellular parts or even additional proteins can participate and modulate the process (see, for example, refs 5, 6). Within the voltage-sensing domain, S4 shows an array of positive charges that drive its movement upon changes in voltage across the membrane^2^,^3^,^7^. Periodically distributed positively charged amino-acid residues at the S4 segment sense changes in the electric field across the membrane, and this stimulus is transduced into a conformational change in the permeation module that finally allows ion flow^4^.

The mechanism responsible for converting the changes occurring in the ‘voltage-sensing module’ into an opening of the gate of the ‘permeation module’ is not well understood. Current knowledge indicates that the intracellular linker between S4 and S5 segments is crucial for the so-called ‘electromechanical coupling’ in potassium channels by forming a rigid α-helical connection^8^,^9^ between the voltage sensor and the pore module^10^,^11^ that acting as a mechanical lever^12^,^13^,^14^ exerts force on the bottom part of S6, constituting the structural link coupling voltage sensing to channel gating^4^,^15^. Most evidence stems from channels of the families *KCNA* to *D*, although some studies have also been performed on HERG (KCNH2, K_V_11.1) (refs 10, 16, 17, 18). In this case, it has been proposed that an electrostatic interaction between the S4–S5 linker and the bottom part of S6 stabilizes the closed conformation, and that outward S4 movement should pull on the linker, subsequently pulling also the S6 bottom resulting in pore opening^10^,^19^,^20^.

We decided to test the relevance of the S4–S5 linker in channels of the KCNH family (K_V_10.1, K_V_10.2 and K_V_11.1) for several reasons. Although these channels lack the PVP motif at the end of S6 that induces a kink that allows channel gating in classical Shaker-like K_V_ channels^21^, trapping of high- and low-affinity blockers upon channel closing^22^,^23^ and cysteine and proline scans of the S6 segment^24^,^25^, indicate that in K_V_11.1 channels the main ion conduction gate (‘activation gate’) is also formed by the bundle crossing of the inner portion of the four pore domain helices. In addition, KCNH channels share a relatively negative activation threshold and are open at 0 mV, raising the possibility that coupling between the voltage-sensing and permeation modules may be required to maintain the channel closed, rather than to open the gate. Finally, K_V_10.1 (*KCNH1*, Eag1) offers an interesting model for the study of voltage-dependent gating, because its activation is slow, permitting relatively easy characterization, but also because the time constant of activation depends very strongly on the prepulse potential and is modulated by extracellular magnesium^26^ allowing an unequivocal identification of its currents. Moreover, the activation/inactivation properties of K_V_11.1 and the implication of different regions of the channel in determining such properties have been studied in great detail, because its peculiar kinetic properties are crucial for the physiological role of K_V_11.1 during the cardiac action potential^27^.

In this report, we demonstrate that at least for KCNH channels, the physical continuity between the voltage sensing and the pore modules is not necessary for voltage-dependent gating, challenging the classical view of a S4–S5 linker acting as a rigid mechanical coupler between them, and opening new questions about the nature of the molecular and functional interactions between the voltage-sensing and pore modules of the protein.

## Results

### Voltage-gated currents in channels split at the S4–S5 linker

**Figure 1:**
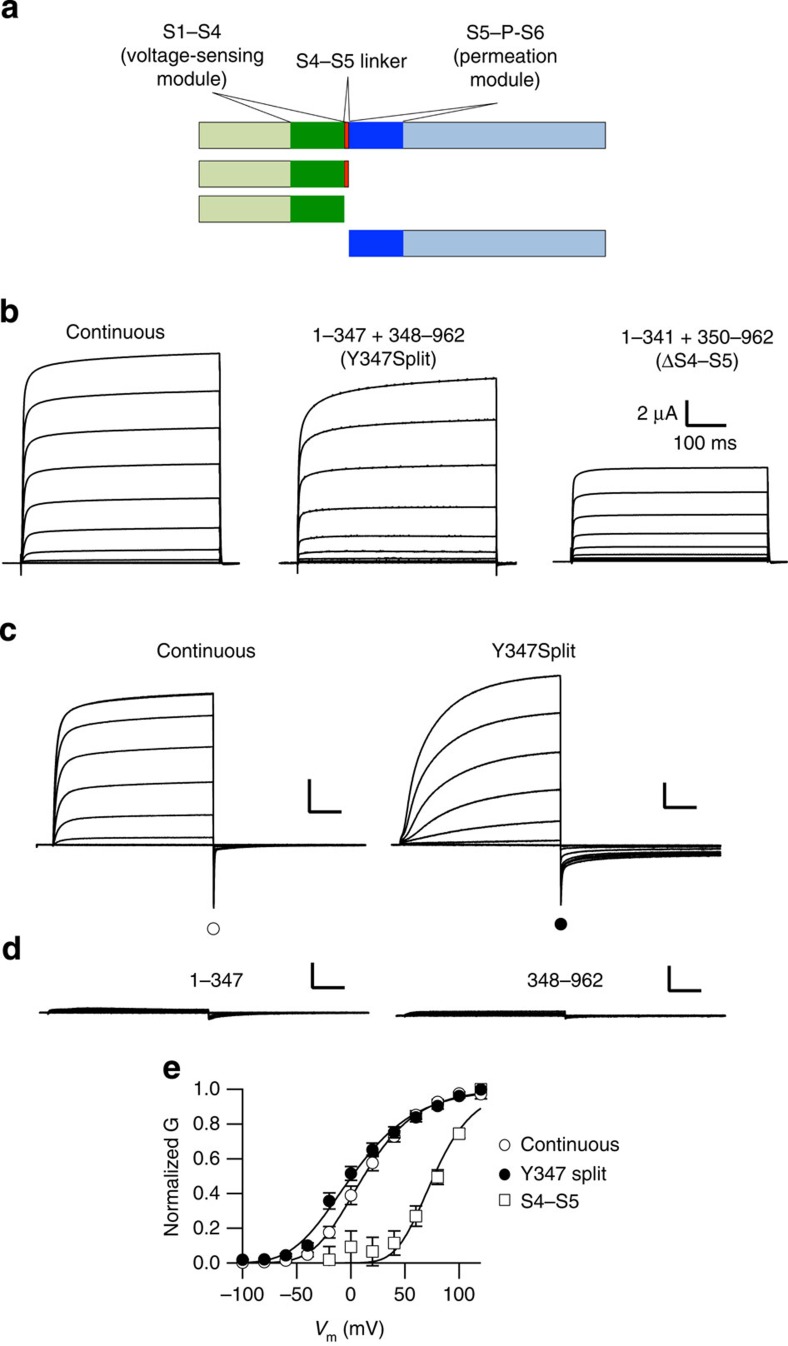
Discontinuous K_V_10.1 channels give rise to voltage-dependent currents. (**a**) Schematic representation of K_V_10.1 deletions. The voltage-sensing domain is depicted in green, the S4–S5 linker in red and the pore module in dark blue. (**b**) Families of current traces obtained by depolarizations to different potential (up to +80 mV at 20 mV intervals) from a holding voltage of −80 mV in oocytes injected with cRNA for continuous K_V_10.1 (left), a mixture of cRNAs encoding for residues 1–347 and 348–962 (centre) or residues 1–341 and 350–962 (right). All gave rise to outward currents with similar kinetics, but with some differences in their voltage dependence as illustrated in **e.** Extracellular solution containing 2.5 mM KCl and no MgCl_2_ was used. (**c**). Representative families of currents for continuous and 347-split K_V_10.1 channels recorded in 60 mM extracellular K^+^. The tail amplitude at time 0 (represented in **e**) was extrapolated from biexponential fits as described in Methods and as indicated by symbols. (**d**) Families of currents elicited in 60 mM extracellular K^+^ using oocytes injected with cRNA encoding 1–347 (left) or 348–962 (right) K_V_10.1 demi-channels. Note the lack of currents after injection of each of the demi-channels alone as compared with the continuous and the split channels (lower panels; scale bars, 2 μA, 100 ms). (**e**). Conductance/voltage plot of continuous (open circles, *n*=13), 347-split (closed circles, *n*=12) and split K_V_10.1 channels lacking the S4–S5 linker (open squares, *n*=11). The currents elicited by channels completely lacking the S4–S5 segment required stronger depolarizations to develop, but the slope (that is, voltage dependence) was not dramatically different. Error bars represent s.e.

To study the requirement of the integrity of the S4–S5 linker, we introduced a stop codon in K_V_10.1 after each residue in the linker (341–349: sequence LDHYIEYGA) and removed the rest of the channel sequence. The truncated proteins were expressed in *Xenopus* oocytes in combination with the carboxyl-terminal (C-terminal) half of the channel, generated by removal of the initial coding sequence and positioning a start codon at the beginning of S5 (before residues 342 to 350, see schematic representation in Fig. 1a). The resulting constructs were transcribed *in vitro* and combinations of two RNAs (encoding together the whole sequence interrupted at each of the positions: for example, 1–347 with 348–962) were co-injected into oocytes. None of the tested constructs gave rise to current when injected alone (Fig. 1d; >20 oocytes measured of each type in at least five independent injections). In contrast, when any of the combinations of interrupted amino (N)- and C-terminal truncated proteins were expressed together (as *split* channels^28^), it was possible to measure voltage-dependent potassium currents. Current amplitudes were smaller in split than in continuous channels (for example, at +40 mV, 12.26±1.27 μA, mean±s.e., *n*=29 in the split versus 21.55±4.58 μA, mean±s.e. *n*=27 for the wild type, when injecting 1 ng RNA), but the difficulties in adjusting RNA concentration per oocyte when injecting two different species at the same time and the requirement of longer incubation periods for robust expression of the split channels prevented a systematic study of crude current amplitudes. Representative current traces of the split channel interrupted after Y347 are shown in Fig. 1b,c in 2.5 and 60 mM extracellular K^+^, respectively, and its voltage dependence is shown in Fig. 1e. The conductance/voltage plots were obtained through extrapolation to time zero of a double exponential fit of the tail current obtained in the presence of 60 mM K^+^ in the external solution (substituting 60 mM Na^+^) as indicated by the corresponding symbols in Fig. 1c. The semi-maximal activation was shifted in the split channel to less depolarized potentials (11.9±0.67 –split, mean±s.d., *n*=8– versus 16.3±0.58 mV –wild type, mean±s.e., *n*=13–) and the slope was shallower (26.2±0.46 versus 20.5±0.30 mV, mean±s.e.). The differences were nevertheless not dramatic, indicating that the combination of the two independent demi-channels results in a functional, voltage-gated ion channel with properties similar to the continuous protein.

Our approach allowed us to combine any of the N-terminal halves with the C-terminal demi-channel starting at position 350, generating *de facto* deletions of 1 to 9 residues in the linker. In the case of the longest deletion (co-expression of demi-channels 1–341 and 350–962), the complex would eventually have no S4–S5 linker. When injected into oocytes, every combination of constructs gave rise to voltage-dependent currents consolidating the concept of a functional coupling between the voltage-sensing module and the permeation module in the absence of physical continuity. This was also true for the split channel completely lacking a S4–S5 linker (Fig. 1b). Although in this case the threshold for activation was strongly shifted to depolarized potentials (Fig. 1e), and the *I*/*V* curves did not reach saturation in the voltage range tested, precluding an accurate estimation of voltage-dependence parameters, there was unequivocal voltage dependence. The activation of this construct was also remarkably slower than wild type, and therefore the accurate estimation of conductance at moderate depolarizations would require very long stimuli. This lack of accuracy at low depolarizations could explain the apparent biphasic behaviour of this construct, but we have not further studied this property.

### Demi-channels coexist at the plasma membrane of oocytes

**Figure 2:**
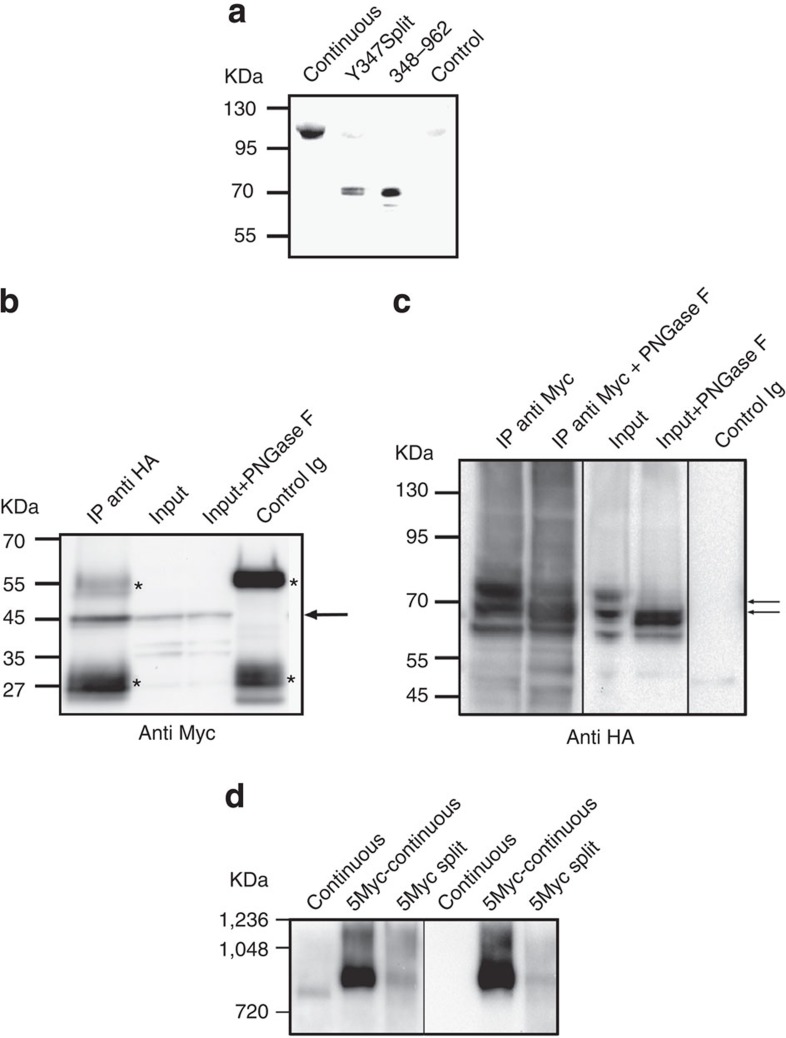
Both K_V_10.1 demi-channels are detected in the same complex. (**a**) Biotinylation of surface proteins and subsequent pull down of labelled molecules allowed the detection of full-length channel using an anti-K_V_10.1 C-terminal antibody, and also of the C-terminal truncated protein when expressed either alone or as split channel. (**b**) Co-immunoprecipitation of N- and C- terminal demi-channels. The N-terminal fragment was labelled with 5 × Myc and the C-terminal was 4 × HA tagged. Immunoprecipitation with HA-tag pulled down a fragment of size compatible with the N-terminal demi-channel (arrow), recognized by anti-Myc immunoblot. Asterisks indicate bands corresponding to the antibody used to immunoprecipitate. (**c**) Immunoprecipitation with anti-Myc also pulled HA-tagged fragments detected as a double band (arrows). The migration distance of the upper band was modified by deglycosylation (PNGase F lanes), as expected for the C-terminal fragment of K_V_10.1, which contains the glycosylated residues. Input lanes were loaded with the extract corresponding to half an oocyte; the equivalent to 30 oocytes were used to immunoprecipitate. (**d**) Native electrophoresis and immunoblot shows the presence of a complex with size similar to that of the continuous channel when demi-channels were expressed together, recognized by both anti-Myc and anti-K_V_10.1 antibodies.

Our results unequivocally show that co-expression of two independent RNAs each encoding a ‘demi-channel’ give rise to voltage-gated currents. This can be due to the assembly of the two independent proteins into multimers that recapitulate properties of the continuous channel, but it could also be possible that expression of both fragments induce the functional expression of an otherwise not detected endogenous voltage-gated channel. However, this cannot be due to only one of the fragments, since we did not observe either outward or tail currents upon injection of any of the demi-channels alone (Fig. 1d). Interestingly, surface biotinylation experiments (Fig. 2a) combined with immunoblot using a polyclonal anti-K_V_10.1 C-terminal antibody to detect only protein exposed to the extracellular medium revealed that the C-terminal demi-channel is indeed expressed on the surface of injected oocytes, not only when injected together with the N-terminal part (second lane in Fig. 2a), but also when injected alone (third lane). As stated below, the results with non-conducting mutant demi-channels also indicate that the currents elicited by co-expression of both channel halves are not due to an endogenous oocyte channel.

To confirm that the two demi-channels actually co-assemble into a single complex and are still independent proteins, we tagged each of them using 5 × Myc (on the N terminus) or a 4 × HA tags (C-terminal) to perform cross pull-down experiments. Pull down of HA gave a signal in Myc immunoblots (Fig. 2b) and vice versa, pull down of the N-terminal fragment (Myc) gave a positive signal in HA western blot (Fig. 2c). In both cases, the pulled-down fragments had the expected size (~50 kDa for the N-terminal and ~70 kDa for the C-terminal demi-channels), indicating that the two halves are not covalently bound in the oocyte. The full-length protein carries sugar moieties in the S5–S6 linker, close to the pore^29^. To test if the C-terminal fragment (HA), which contains the glycosylation sites, could also be glycosylated in the split channels, we performed enzymatic deglycosylation of oocyte extracts (Fig. 2c). Treatment with PNGase F induced changes in the electrophoretic mobility of the HA-positive band, compatible with complex glycosylation of the truncated protein. The same treatment did not induce changes in the migration pattern of the band detected by Myc, which is not expected to carry glycosylation (Fig. 2b).

Finally, when extracts from oocytes were run under native conditions, co-expression of the two fragments gave rise to a large complex, with migration similar to the full-length channel (Fig. 2d). This again indicates that both fragments are expressed and share the same complex.

In summary, we conclude that upon expression of the channel fragments, a functional complex is formed by the apposition of voltage sensors and permeation modules that gives rise to a functional, voltage-dependent ion channel.

### Split channels retain the properties of both functional modules

**Figure 3:**
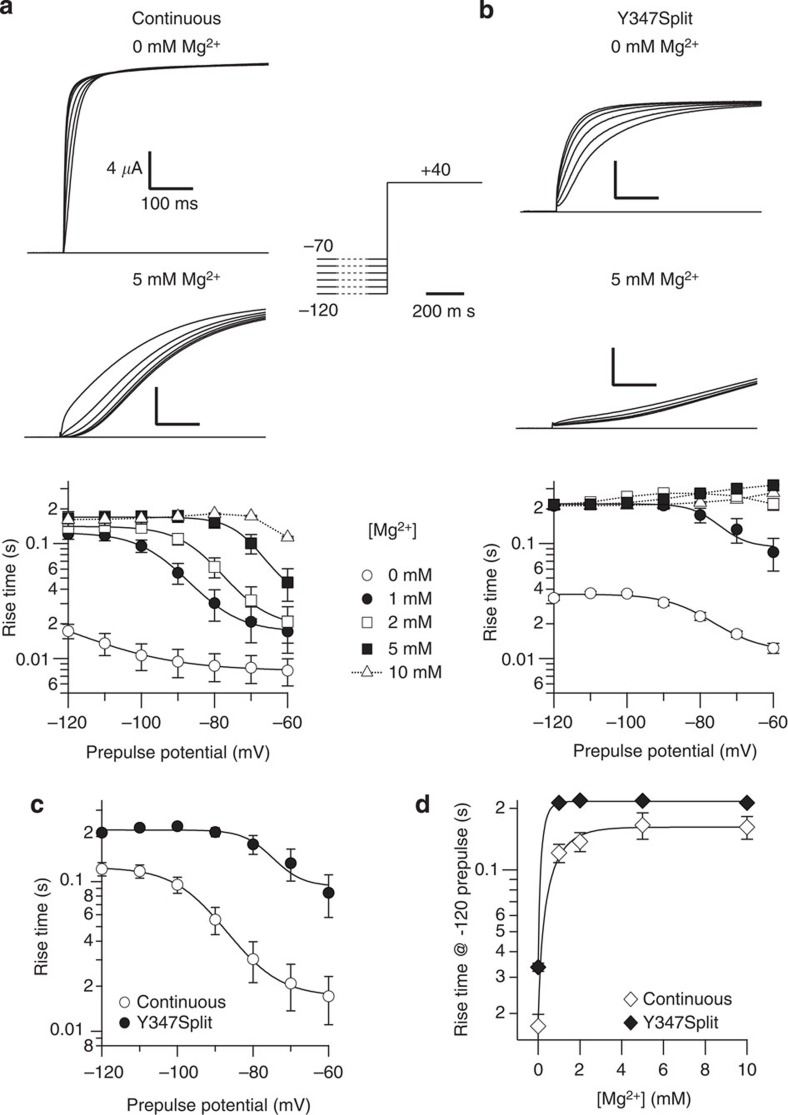
Split channels retain dependence on the prepulse potential and on extracellular Mg^2+^. (**a**,**b**) Representative current traces obtained following the depicted protocol, holding the oocyte for 5 s (indicated in the schematic as a dotted line) at the indicated potential and immediately stimulating to +40 mV in the absence (upper traces) or the presence (middle traces) of 5 mM extracellular Mg^2+^. Both wild-type (**a**) and split (**b**) channels show accelerated activation at less hyperpolarized potentials. Also in both cases, Mg^2+^ slows down the activation. The effect of prepulse potential at different Mg^2+^ concentrations is depicted in the lowest panels (*n*=5–8). Over 1 mM, the activation of the split channel is so slow that the dependence on prepulse is not any longer evident. The solid lines indicate fits to sigmoid functions, dotted lines represent polynomial fits. (**c**) Comparison of data (from **a**,**b**) of continuous and split channel in 1 mM Mg^2+^. (**d**) Effect of Mg^2+^ concentration on the speed of activation of continuous and split channels. The activation was slower in the split channels (closed symbols) than in control (open symbols) under all conditions tested. Error bars represent s.e.

If the demi-channels indeed form a voltage-gated complex, properties typical of both voltage sensor and pore modules should be conserved in the split channel. A defining feature of K_V_10.1 is its high sensitivity to the prepulse potential^30^. The channel activates faster the more depolarized the potential before the stimulus (Fig. 3), and this phenomenon is strongly dependent on extracellular magnesium through interaction with residues located in the voltage-sensing module of the channel^31^. Moderate concentrations of Mg^2+^ slow down the activation and make the phenomenon most evident (Fig. 3a). The activation of the split channel was slower under all experimental conditions, but the dependence on the prepulse potential was preserved, as depicted in Fig. 3b. A comparison between the continuous and the split channel in the presence of 1 mM extracellular Mg^2+^ is shown in Fig. 3c. Over 1 mM, the activation of the split channel was so slow that the changes induced by hyperpolarized prepulses became less obvious (Fig. 3d). In summary, the split channel maintains both the dependence on the prepulse potential and dependence on extracellular Mg^2+^.

**Figure 4:**
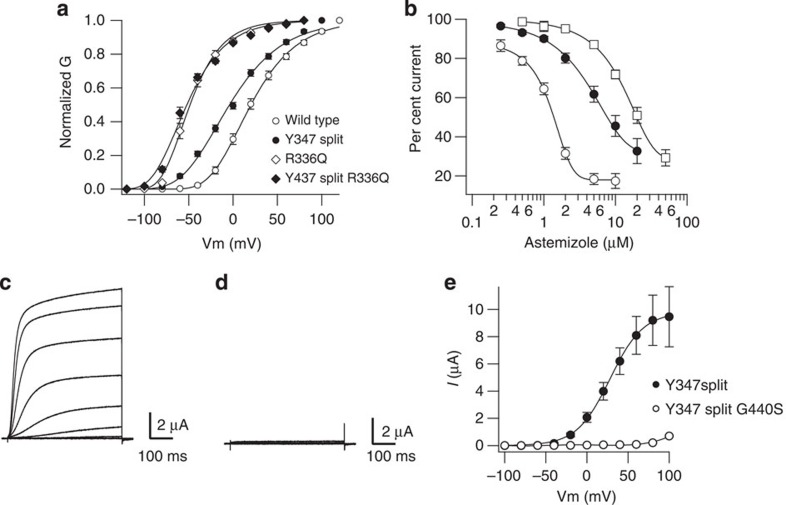
Split channels retain properties residing in both the voltage-sensing and permeation modules. (**a**) Mutation of the voltage-sensor domain has similar effects in continuous and split channels. Neutralization of a positive charge in the S4 domain (R336Q) shifts the activation potential to hyperpolarized values regardless of the integrity of the S4–S5 linker (Open circles, wild type; closed circles, split channel; open diamonds, R336Q; closed diamonds, split R336Q; *n*=7; error bars, s.e.). (**b**) Blockade by astemizole, whose structural determinants lie in the C-terminal part of the protein, is conserved both in channels with interrupted S4–S5 (closed circles, *n*=6–7) or lacking completely the linker (squares, *n*=2–4), albeit with reduced affinity as compared with control (open circles, *n*=9–16). (**c**–**e**) A pore mutation precluding K^+^ permeation abolishes the activity also of split channels (**c**, wild-type split; **d**, G440S mutant split) (**e**) *I*/*V* relationship of wild-type and mutant split channels constructed with values obtained from 10 oocytes. Error bars represent s.e.

To further confirm that the activation of the split channel still depends on the voltage-sensor domain, we co-expressed a mutated N terminus where the voltage dependence had been shifted by neutralization of one of the positive charges in S4 (R336Q) with the wild-type C-terminal module. The resulting split channel displayed a shift of voltage dependence with respect to the wild-type split (Fig. 4a) comparable to the one observed in the continuous channel, strongly indicating that the voltage dependence observed in the split channel is indeed conferred by the properties of the voltage-sensing module.

To probe the properties of the permeation module of the split channel, we used astemizole, which is a relatively well-studied blocker of channels of the KCNH family^32^. The crucial residues for astemizole block lie in the C-terminal half of the channel (F468) (ref. 33), and we expected them to be largely conserved in the split channel. Indeed, astemizole still blocked both the split and the channel without a S4–S5 linker, although the IC_50_ was shifted to the right in both cases (Fig. 4b). In addition, we generated a split channel carrying a mutation in the deep pore of K_V_10.1 that abolishes permeation (G440S)^34^. This combination did not give rise to detectable currents in oocytes (Fig. 4c–e), further indicating that the currents detected in the presence of the split are not due to endogenous overproduced oocyte channels. Fig. 4e shows the average current–voltage relationships obtained from 10 oocytes in those experiments.

Altogether, the expression of K_V_10.1 as two independent proteins containing the voltage-sensing and the permeability module, respectively, generated currents recapitulating properties attributable to either module, strongly indicating that the detected current depends on the association and interaction of both independent proteins, that correctly assemble and generate functional channels in the absence of physical continuity between them.

### Functional expression of other S4–S5 split KCNH channels

**Figure 5:**
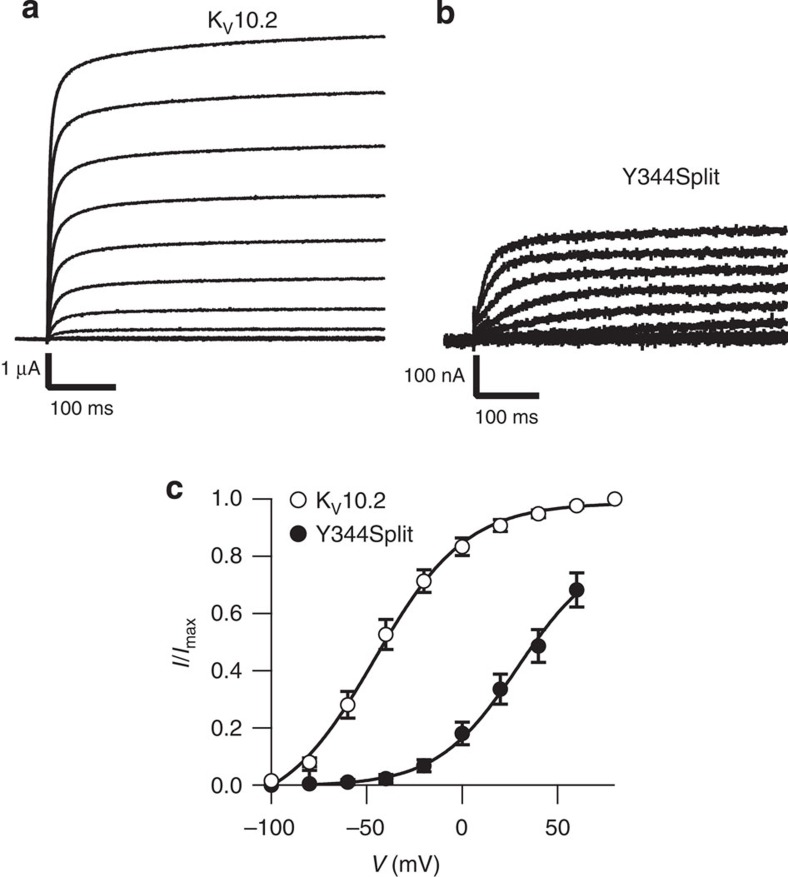
Interruption of the S4–S5 linker renders also functional K_V_10.2 channels. Current amplitudes were always bigger in continuous (**a**) than split (**b**) channels. The split channel (solid symbols, *n*=4 for this plot) also required larger depolarization than the continuous channel (open symbols, *n*=14) to be activated (**c**). Error bars represent s.e.

A relevant question is whether our observations respond to a peculiar behaviour of K_V_10.1 or are rather extendable to other channels. We therefore tested two other KCNH members, K_V_10.2 (Eag2, encoded by *KCNH5*) and K_V_11.1 (HERG, encoded by *KCNH2*). K_V_10.2 shares high homology with K_V_10.1, and was truncated at Y344, the position equivalent to Y347 of K_V_10.1. Expression of K_V_10.2 wild type (Fig. 5a) is in our hands much less efficient than that of K_V_10.1, and so was also the case for the split channel (Fig. 5b). The conductance/voltage plot of the K_V_10.2 split channel showed a marked shift to more positive potentials and thereby lost the very negative activation threshold of the parental channel (Fig. 5c). Nevertheless, we could observe potassium currents compatible with K_V_10.2 in oocytes injected with the two truncated proteins.

**Figure 6:**
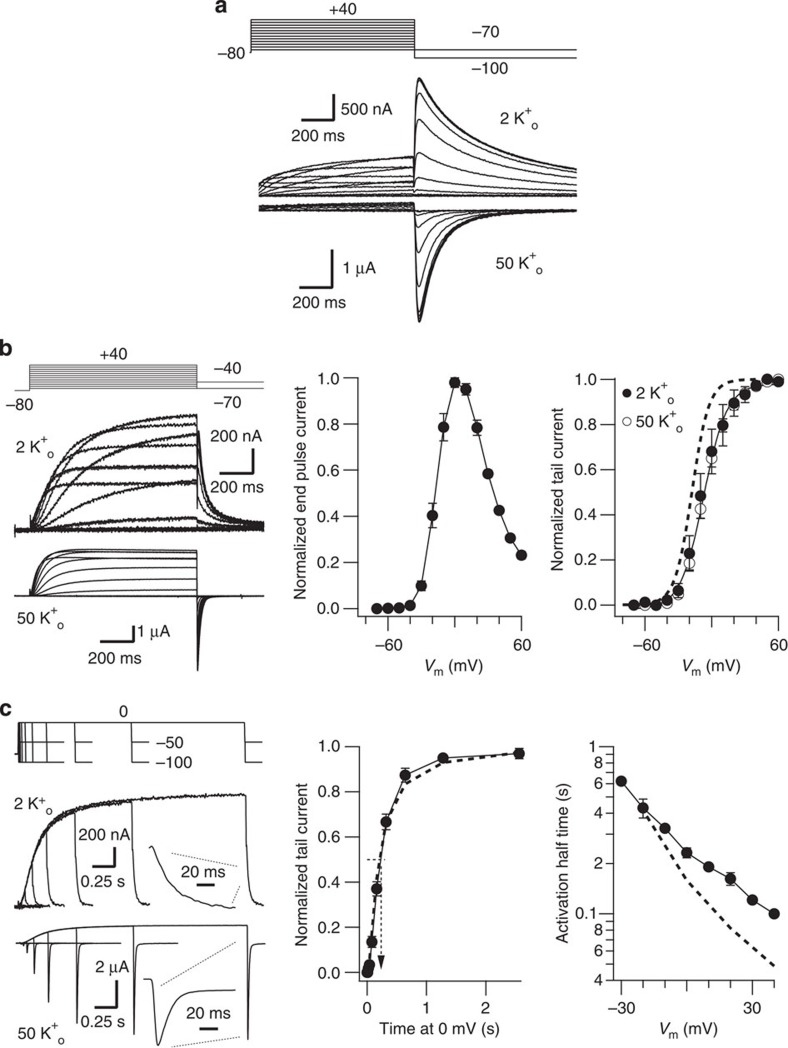
Functional expression and activation kinetics of continuous and Y545 Split K_V_11.1 (HERG) channels. (**a**) Representative current traces of continuous wild-type HERG channels in response to the protocol shown at the top. Extracellular medium containing 2 and 50 mM KCl was used as indicated. (**b**) Voltage-dependent activation of Y545 Split K_V_11.1 channels. Left, raw current traces recorded in 2 and 50 mM extracellular K^+^ in response to 1 s depolarization pulses between −70 and +60 mV at 10 mV intervals from a holding potential of −80 mV, followed by a repolarizing step to −40 (2 mM K^+^) and −70 (50 mM K^+^) mV. Centre, averaged *I* versus *V* relationship measured at the end of the depolarization step in 2 mM external K^+^ (*n*=8). Note the typical n-shaped curve due to the strong rectification as a result of the typical K_V_11.1 slow activation and fast inactivation overlap at positive voltages. Right, plots of normalized peak tail currents as a function of depolarizing voltage in 2 (closed circles) and 50 mM (open circles) extracellular K^+^ (*n*=13). A curve from wild-type continuous K_V_11.1 channels obtained in the same conditions is shown as a dashed line for comparison. (**c**) Y545 Split K_V_11.1 voltage-dependent activation rates. Left, time course of current activation at 0 mV in 2 (top traces) and 50 mM (lower traces) extracellular K^+^. The duration of a depolarizing prepulse from a holding potential of −80 mV was varied and followed by a repolarization step to −50 and −100 mV in 2 and 50 mM external K^+^, respectively. An enhanced view of the tail currents at the end of the 2,560 ms depolarizing steps is shown in the insets. Centre, plot of normalized tail current magnitude versus depolarization time at 0 mV (*n*=4). The plot was used to measure time necessary to attain half-maximum current magnitude (dashed arrow). Note the sigmoidal nature of the early activation time course during the initial tens of ms. Right, dependence of activation rates on depolarisation membrane potential (*n*≥4). Values from wild-type non-split channels are shown as a dashed line for comparison. Error bars represent s.e.

**Figure 7:**
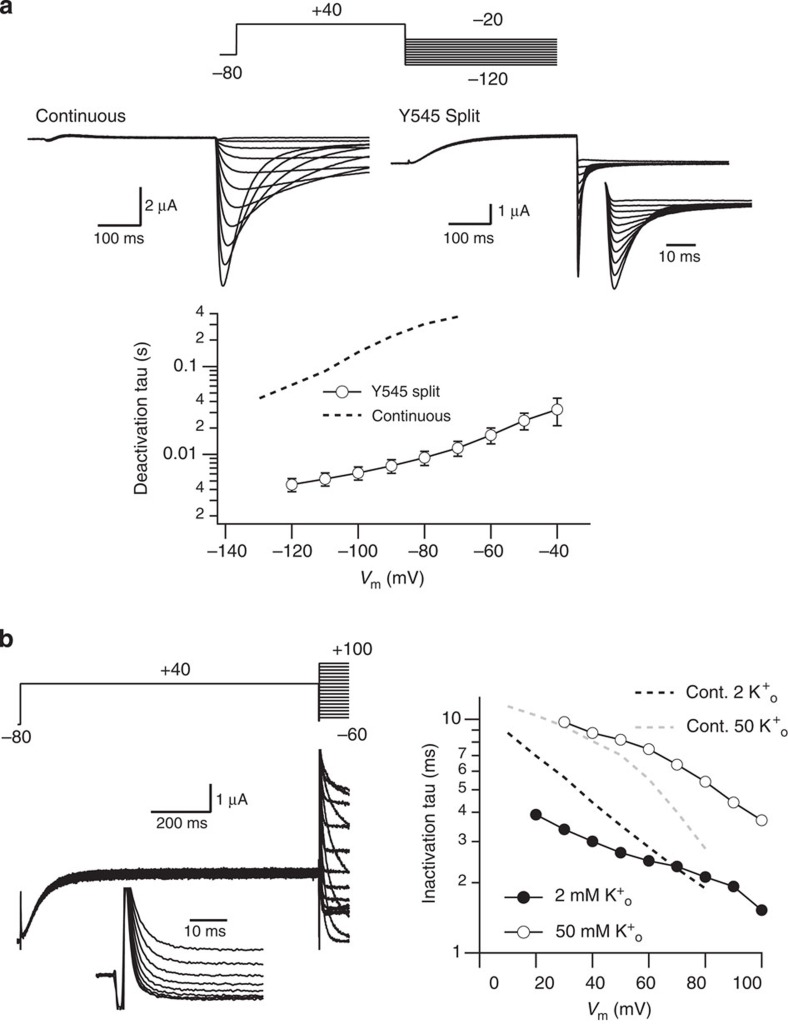
Characterization of deactivation and inactivation properties of Y545 Split K_V_11.1 channels. (**a**) Voltage-dependent deactivation. Top panels, representative current traces recorded in 50 mM extracellular K^+^ during steps to potentials ranging from −20 to −120 mV at 10 mV intervals, following depolarizing pulses to +40 mV from a holding potential of −80 mV as schematized at the top. Currents from oocytes expressing continuous wild-type (left) and Y545 Split K_V_11.1 (right) are shown for comparison. For the split, also an enhanced view of the peak tail currents during the repolarization steps is shown in the inset. Lower panel, plot of fast deactivation time constant for different repolarization voltages for split (open circles, *n*=4) and wild-type non-split channels (dashed line). Error bars, s.e. (**b**) Measurement of Y545 Split K_V_11.1 inactivation rates. Left panel, onset of fast inactivation at different voltages was studied in 2 mM extracellular K^+^ with the triple pulse protocol shown at the top, in which the channels were activated and inactivated with a 1 s prepulse to +40 mV, followed by a second short prepulse to −100 mV to recover the channels from inactivation and a test pulse to different voltages from −60 to +100 mV at 10 mV intervals to reinactivate them. Membrane currents starting at the end of the depolarizing prepulse for depolarizations between +20 and +100 mV at which the inactivation and deactivation kinetics barely overlap, are shown in the inset. Right panel, plot of time constants for the onset of inactivation in 2 mM (closed circles) and 50 mM (open circles) extracellular K^+^ as a function of voltage, obtained from single-exponential fits to the decaying portion of the currents during the test pulses. Values from wild-type non-split channels at the same K^+^ concentrations are shown as black (2 mM) and grey (50 mM) dashed lines for comparison.

K_V_11.1 (HERG) could also be expressed as combination of two demi-channels: an initial half truncated after Y545, in the middle of the putative S4–S5 linker, thus corresponding to residues 1–545, and a truncated channel covering residues 546-1159. Voltage-evoked currents reminiscent of those of continuous K_V_11.1 (Fig. 6a) were obtained upon expression of the split HERG. Thus, while the magnitude of the wild-type peak tail current measured at −50 mV in 2 mM extracellular K^+^ amounted 1.68±0.32 μA (mean±s.e., *n*=26, *N*=11), the Y545 split peak tail current reached 0.36±0.06 μA (mean±s.e., *n*=19, *N*=9). This value increased to 0.6±0.1 μA (mean±s.e.) when the tail current magnitude was estimated by extrapolation at zero repolarization time to prevent the reduction in the peak current imposed by the very fast deactivation decay of the split tails (see below). As expected, no detectable currents were observed when any of the demi-channels were separately expressed in the oocytes (Supplementary Fig. 1). The split channel currents exhibited the typical K_V_11.1 inward rectification at positive voltages. The voltage dependence of the steady-state activation was only slightly shifted to positive potentials (Fig. 6b). Also, the voltage-dependent activation rate of the split was only slightly slower than wild type at positive voltages (Fig. 6c). Alterations in the S4–S5 loop of K_V_11.1 are expected to produce an acceleration of deactivation^35^. Consistently, the deactivation time constant of the discontinuous channel was accelerated by an order of magnitude (Fig. 7a). Finally, the inactivation of the split K_V_11.1 was also similar to the wild-type channel (Fig. 7b) and appeared clearly slowed when the extracellular K^+^ level was raised from 2 to 50 mM (refs 27, 36). Thus, co-expression of the two demi-channels rendered currents with the predicted properties, further indicating that the S4–S5 linker is indeed interrupted, and that there is no covalent fusion of the two proteins during synthesis, assembly and/or trafficking.

**Figure 8:**
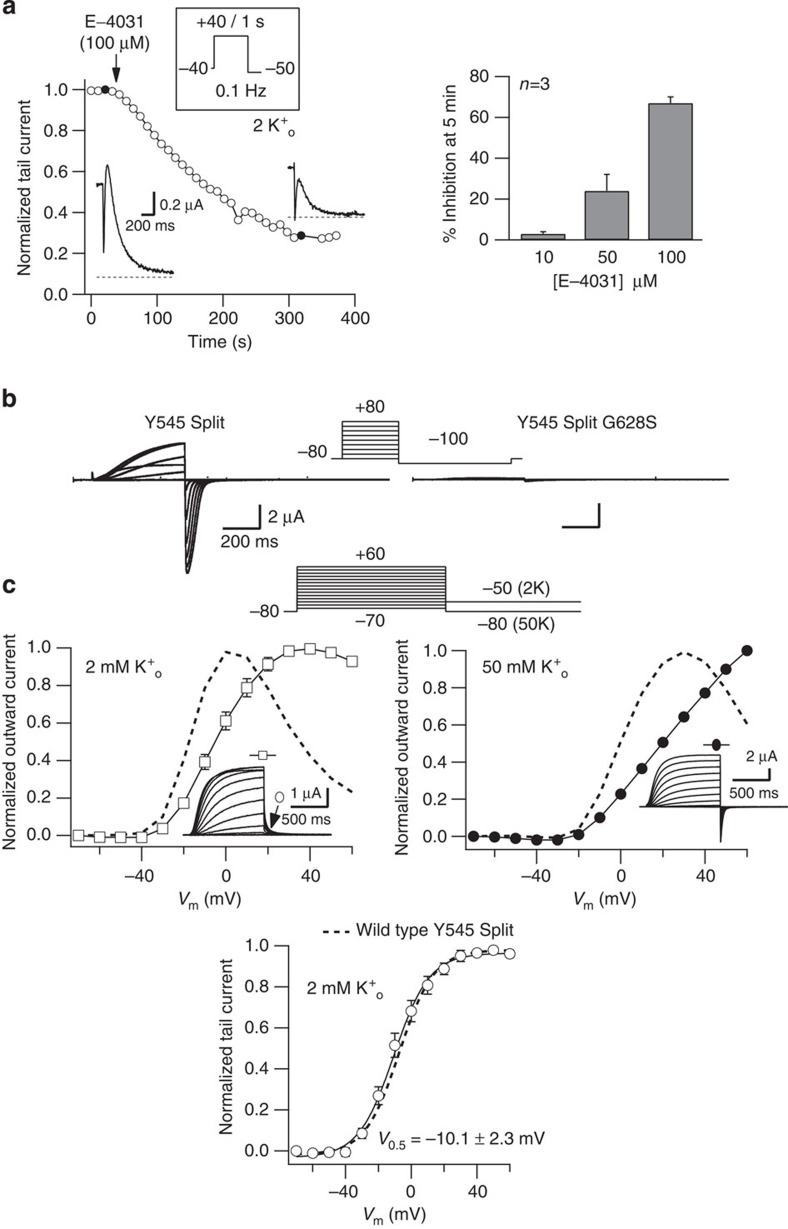
Y545 Split HERG channels retain properties residing in the permeation module. (**a**) Inhibition of split HERG currents by E-4031. The time course of current inhibition in response to an addition of 100 μM E-4031 is shown on the left. Currents were elicited in response to 1 s depolarizing pulses to +40 mV from a holding potential of −40 mV followed by a repolarization step to −50 mV. Pulses were delivered at a frequency of 0.1 Hz. Representative tail current traces before and after 5 min of treatment with E−40131 are shown on the insets. A bar histogram relating the extent of current inhibition with the concentration of the inhibitor is shown on the right. (b). Absence of functional expression upon co-injection of cRNAs encoding N-terminal 1–545 and non-conducting C-terminal 546–1,159 K_V_11.1 demi-channels carrying a G628S point mutation in the pore. Currents were obtained in 50 mM extracellular K^+^ using the voltage protocol shown at the top. Families of currents from non-mutant (left) and G628S (right) splits are shown. (**c**) Characterization of voltage-dependent currents elicited by co-expression of K_V_11.1 demi-channels carrying a S620T point mutation in the pore domain. The voltage protocol used to elicit the currents is shown at the top. Representative families of currents recorded in response to the protocol are shown in the insets. Top panels, *I* versus *V* relationships from normalized currents measured in 2 mM (open squares, left) and 50 mM (closed circles, right) extracellular K^+^ at the end of the depolarization step. Lower panel, current/voltage relationship generated using peak tail currents measured in 2 mM extracellular K^+^ (open circles). Data from normally inactivating non-mutated split channels obtained in the same conditions are shown as dashed lines (Y545 Split) for comparison. Error bars represent s.e.

**Figure 9:**
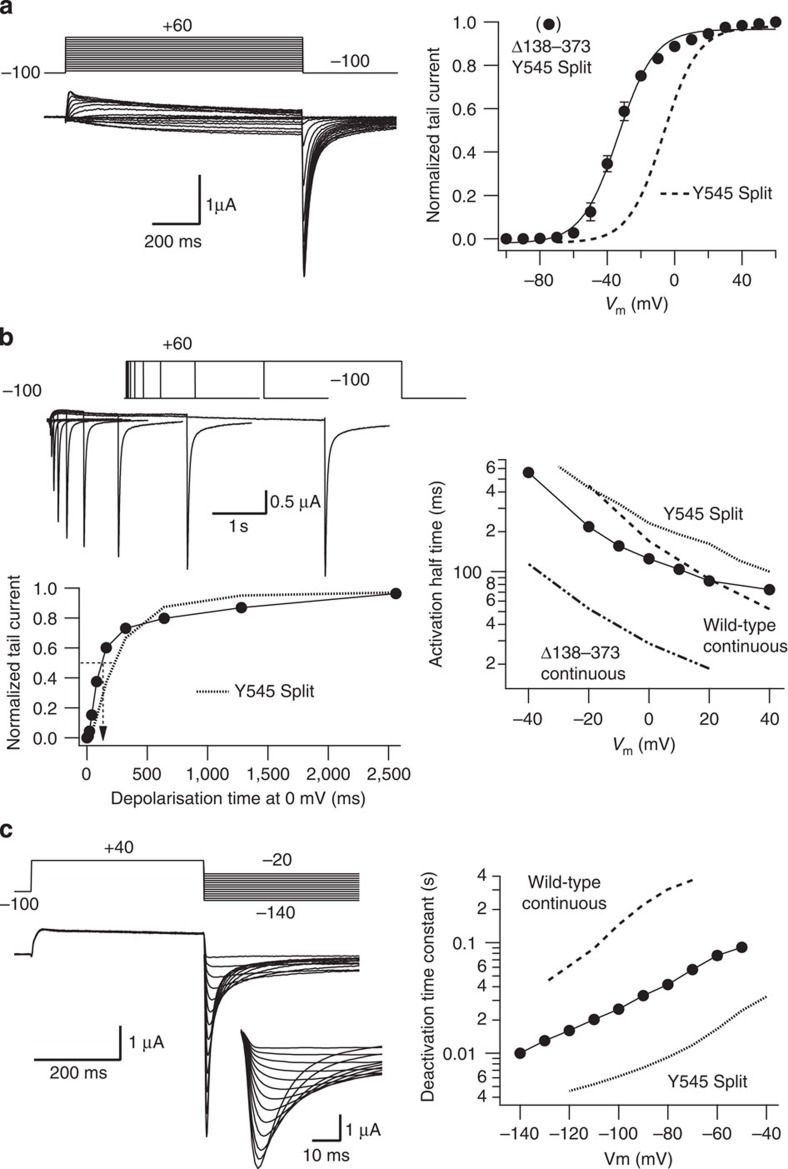
Characterization of voltage-dependent currents elicited by co-expression of K_V_11.1 demi-channels carrying a Δ138–373 deletion. (**a**) Voltage-dependent activation of K_V_11.1 C-terminal and N-terminal demi-channels carrying a Δ138–373 deletion corresponding to the proximal domain located at the cytoplasmic segment, preceding helix S1 in the initial half of the voltage sensor module. Left panel, raw current traces recorded in 50 mM extracellular K^+^ in response to the voltage protocol shown at the top. Right panel, plot of normalized peak tail currents as a function of depolarizing voltage. A curve from non-deleted split channels obtained in the same conditions is shown as a dotted line for comparison. Error bars represent s.e. (**b**) Determination of voltage-dependent activation rates. Left panels, time course of current activation at 0 mV studied in 50 mM extracellular K^+^. Representative currents recorded in response to the illustrated voltage protocol are shown at the top. A plot of normalized peak current value versus depolarization time at 0 mV is shown at the bottom. The time necessary to attain half-maximum current magnitude is marked with a dashed arrow. Right panel, dependence of activation rates on depolarization membrane potential. Values from wild-type non-split channels (Wild type continuous), non-deleted split channels (Y545 Split) and non-split channels lacking the proximal domain at the amino terminus (Δ138–373 continuous) are also shown for comparison. (**c**) Voltage-dependent deactivation. Left panel, representative current traces recorded in 50 mM extracellular K^+^ during steps to potentials ranging from −20 to −140 mV with the voltage protocol schematized at the to*p*. An enhanced view of the peak tail currents during the repolarization steps is shown in the inset. Right panel, plot of fast deactivation time constant for different repolarization voltages. Values from wild-type non-split channels (wild-type continuous) and non-deleted split channels (Y545 Split) are also shown for comparison.

Similar to the results observed with the split K_V_10.1 channel, the currents elicited after co-expressing the two truncated HERG halves were sensitive to E-4031 (Fig. 8a), a very specific blocker of HERG^37^, although with lower affinity than the intact channel^38^. Furthermore, a permeation-inhibiting mutation (G628S)^39^,^40^ equivalent to the one reported for K_V_10.1 in Fig. 4 also abolished functional expression of the split HERG channel (Fig. 8b). Introduction in the split C-terminal permeation module of a S620T pore domain point mutation, known to antagonize K_V_11.1 inactivation^27^, reduced (at 2 mM K^+^) or eventually abolished (in 50 mM) inactivation of the split channel (Fig. 8c), without affecting the voltage dependence of activation (Fig. 8c) or deactivation (Supplementary Fig. 2). Finally, deletion of the N terminus proximal domain (residues 138–373; see ref. 41), which shifts the activation voltage dependence towards more negative values and accelerates activation of HERG channels, induced similar effects when performed in the split N-terminal half of the channel (Fig. 9 and refs 35, 41). In conclusion, the voltage sensor of the initial half is able to confer near normal voltage-dependent properties to the assembled construct, and the functional properties of the pore domain leading to the characteristic voltage-dependent inactivation of HERG are maintained in the co-assembled channels. Altogether our data unequivocally demonstrate that the permeation properties of the split channels are determined by the C-terminal domain, but it is the initial voltage sensor-containing module what crucially determines the voltage-dependent properties of the assembled protein.

### Hybrid split channels from different KCNH family members

**Figure 10:**
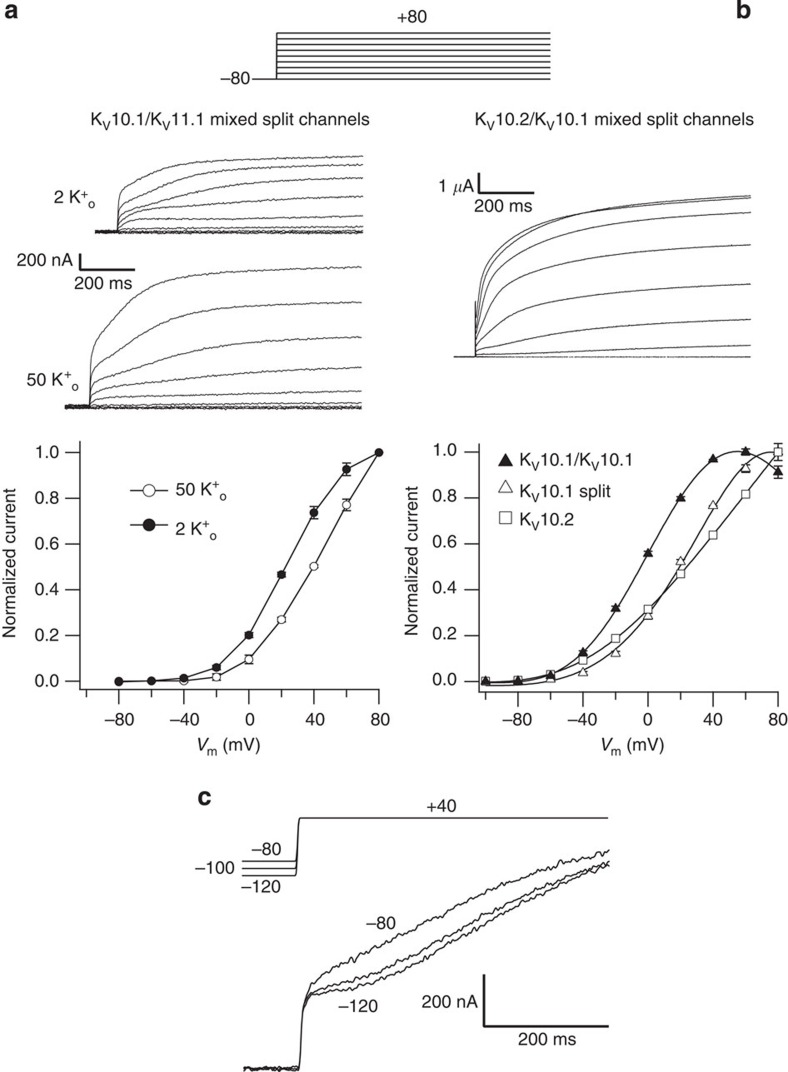
Hybrid split channels yield voltage-dependent currents. (**a**) Voltage-dependent activation of the K_V_10.1 N-terminal/K_V_11.1 C-terminal assembled demi-channels. Current traces as result of 500 ms depolarizations to potentials ranging between −80 and +80 mV in 20 mV intervals in both 2 and 50 mM extracellular K^+^ are shown at the top. The normalized *I*/*V* relationships at both potassium concentrations are shown at the bottom. (**b**) Voltage-dependent activation of the K_V_10.2 N-terminal/K_V_10.1 C-terminal assembled demi-channels. Representative current records in 2 mM extracellular K^+^ are shown. Note the much higher current level obtained with this mixture as compared with **a**. Normalized *I*/*V* relationships of the mixed demi-channels (*n*=18) as compared with K_V_10.1 demi-channels (*n*=23) and K_V_10.2 continuous channels (*n*=17) are shown at the bottom. Error bars represent s.e. (**c**). Acceleration of current activation (‘Cole–Moore’ shift) by holding the K_V_10.1 N-terminal/K_V_11.1 C-terminal mixed demi-channels at more depolarized potentials. The traces correspond to representative currents obtained from oocytes held at the indicated holding potentials upon stimulation to +40 mV using the protocol represented at the top.

To check for the specificity of the split channel assembly, we tried combinations of the N- and C-terminal halves from K_V_10.1 and K_V_11.1 as well as K_V_10.1 and K_V_10.2. No active channels were recorded when the voltage sensor-containing N-terminal half of K_V_11.1 was co-expressed with the C-terminal half of K_V_10.1. However, co-injection in the oocytes of the N-terminal voltage-sensing module of K_V_10.1 (corresponding to residues 1–347, this last one in the middle of the S4–S5 linker) with the C-terminal permeation module of HERG (from residues 546 in the S4–S5 loop to 1,159 at the C terminus) yielded voltage-dependent currents that basically recapitulated those obtained with the K_V_10.1 split (Fig. 10a). No HERG-type inward rectification at positive voltages was observed with this combination, and only a little nonlinearity of the I/V relationship at voltages positive to +40 mV was obtained in 2 mM extracellular K^+^, that was virtually abolished by raising the concentration of the cation to 50 mM. Similarly, the voltage-sensing domain of K_V_10.2 was able to generate functional channels when combined with the pore module of K_V_10.1 (Fig. 10b). Furthermore, the speed of activation of the K_V_10.1/K_V_11.1 combination during the depolarization pulses became faster when the holding potential was maintained at more depolarized values (Fig. 10c), a typical signature of K_V_10.1 (Fig. 3) and K_V_10.2 channels^30^ that is not exhibited by K_V_11.1 (ref. 41). This demonstrates not only that the ability to voltage-dependent gate the HERG pore is maintained upon heterologous co-assembly with the K_V_10.1 voltage sensor in the absence of physical continuity between them, but also that the voltage-dependent properties are mainly conferred by the voltage-sensor moiety of the split. Finally, the truncation of channels of the KCNA (K_V_1.4) or KCNQ (K_V_7.2/3) families did not produce functionally active channels. As discussed below, the reason(s) for this differential behaviour remains to be established.

## Discussion

Our data demonstrate for the first time that the covalent link between voltage sensor and pore module is not necessary to confer voltage-dependent gating properties to KCNH channels. Our results challenge the classical view of voltage-dependent channel activation in which the outward movement of the S4 voltage-sensor helix tracks the S4–S5 linker that acting as a rigid mechanical lever pulls apart the N-terminal portion of S5 and the C-terminal end of S6 lining the channel gate to open it. Such a mechanism would be incompatible with voltage dependence in a split KCNH channel. What molecular system could be involved in coupling the voltage sensor to the pore module in the presence of a structurally interrupted S4–S5 linker? One possibility is that the C-terminal end of S4 and/or the initial half of the S4–S5 linker pushes the S5–S6 module to maintain the gate closed at rest, whereas its movement upon depolarization allows for passive relaxation of the pore module to its default conducting conformation^42^. Unlike Shaker-like K_V_ channels^43^, an intrinsically more stable open state has been proposed for K_V_11.1 (refs 4, 10, 35, 44). However, our data indicate that when injected alone the C-terminal domain does not give rise to detectable potassium currents, even though it is expressed at the plasma membrane, as suggested by the dominant negative effect of the non-permeant demi-channel modules, and directly demonstrated by the biotinylation experiments in the case of K_V_10.1. Further experiments would be necessary to know if this absence of permeability with the isolated pore module is due to a closed conformation of the pore of the demi-channel, a collapse of the pore loops, aggregation or misfolding of the protein in the absence of the other parts of the channel.

A second alternative to explain the functional integrity of the splits is that the end of S4 and/or the beginning of the S4–S5 loop are used to transmit a conformational non-covalent coupling of the voltage sensor movement to opening and closing of the gate at the bundle crossing of S6 (ref. 45). In this case, the interaction between these two structures would transfer the energy generated by S4 movement, to push or pull the opening of the S6-limited gate^15^. Indeed, previous work both in K_V_11.1 (ref. 10) and other Shaker-like K_V_ channels^11^,^12^,^46^ identified a direct interaction between the S4–S5 linker and the C-terminal portion of the S6 helix as a crucial component of the gating process. It has been proposed that this intrasubunit interaction is further complemented by a intersubunit interaction between the lower S4 and the S5 of the neighbouring subunit to drive the final cooperative gating transition leading to pore opening^47^,^48^,^49^. In this context, our results identifying the C-terminal end of S4 and/or the initial segment of the S4–S5 loop (but not the integrity of this linker) as a crucial component of the gating mechanism, point to the possibility that these type of interactions are also involved in voltage-dependent gating of the KCNH channels. Interestingly, a nearly unaltered functionality in channels carrying a cleaved backbone between the voltage-sensing and the pore domains would be consistent with the recently recognized need of a flexible N-terminal segment in the S4–S5 linker for proper K_V_11.1 channel gating^19^,^20^. Altogether, this indicates that instead of a classical electromechanical coupling based in an S4–S5 loop acting as a rigid mechanical lever, at least for KCNH channels the coupling of the voltage-sensing and pore modules is based in an ‘electrointeractional’ mechanism probably involving the end of S4 and/or the beginning of the S4–S5 loop as well as the C-terminal portion of the S6.

The currents observed with the splits are very unlikely due to proteins other than the injected channels. Tagged K_V_10.1 demi-channels are able to immunoprecipitate the counterpart in both directions, that is, precipitating the N terminus pulls down the C-terminal part and vice versa. Furthermore, the split channel is not only detectable on the surface of the oocyte, but it also shows glycosylation resembling the pattern obtained for the continuous channel. The C-terminal demi-channels are detected in native gels in a complex with a size compatible with association with the N-terminal part. These results indicate that both demi-channels associate to form a functional potassium channel.

It is important to note that although maintenance structural integrity of the S4–S5 loop is not strictly necessary to confer voltage-dependent gating, our results also indicate the existence of subtle differences in the behaviour of the splits, that can be aggravated upon complete removal of the linker (see, for example, Fig. 1d). The overall current amplitude was reduced in all channels tested as compared with the respective continuous channel. In addition, splitting induces some alterations in voltage dependence and, at least at certain voltages, a slower activation rate both for K_V_10.1 and K_V_11.1 (Figs 3, 5). Also, the modifications in gating properties induced by removal of the K_V_11.1 N-terminal proximal domain, are less prominent in the split than in the continuous channel (Fig. 9 and refs 35, 41). These observations suggest that the assembly of channels is less efficacious and, once assembled, the communication between the voltage sensing and the permeation modules is altered to a certain extent. On the other hand, some changes in deactivation were observed in split channels of both K_V_10.1 and 11.1. Thus, the marked acceleration of the K_V_11.1 split closing indicates that the S4–S5 segment is also relevant for deactivation, but that its exact role is probably determined by interactions with other parts of the protein, as repeatedly reported for this channel^18^,^50^,^51^,^52^,^53^. This can be expected from the recognized role of the S4–S5 linker as a coupler of voltage sensing to gate, but also as an integrator of cytoplasmic signals that modulate activation and deactivation gating^9^. Indeed, a direct interaction of some intracellular channel structures with the S4–S5 domain has been demonstrated previously (reviewed in ref. 5). In other words, our results indicate that although not completely necessary for voltage-dependent gating, the S4–S5 segment still plays a relevant role for proper function also in KCNH channels. Previous studies with K_V_11.1 carrying mutations at residue 540 or after crosslinking this position to the end of helix S6 (refs 10, 35) indicate that the N-terminal end of the S4–S5 linker plays a crucial role in coupling voltage sensing to channel gating. A similar mechanism may be involved in voltage-dependent coupling of the splits. However, the rest of the S4–S5 linker, probably through interaction(s) with other parts of the channel (for example, the N-terminal eag/PAS and/or proximal domains, or the C-linker immediately below the gate^9^,^17^,^19^,^20^,^41^,^50^,^51^,^52^,^53^) can also strongly influence the properties and extent of such coupling. The reduced voltage sensitivity of split channels lacking a S4–S5 linker would reinforce this view.

Importantly, properties attributable to each part of the full channel, voltage-sensing domain and pore domain, are transferred to the corresponding split channels. Pore blockers such as astemizole and E-4031 inhibit currents through K_V_10.1 and HERG split channels, respectively; pore mutations in both the channels abolish permeation also in the split channels; mutation of the S4 segment induces shift in the same direction in both split and continuous channels and K_V_10.1 split channels retain a marked Cole–Moore shift. Furthermore, Cole–Moore shift, which is not detected in HERG channels, is conferred to K_V_10.1/K_V_11.1 hybrid split channels by the voltage-sensing domain of K_V_10.1, reinforcing the concept of modularity of ion channels.

Up to now, the results obtained with the split KCNH channels were not reproduced with members of the *KCNA* or *KCNQ* families. This could be attributed to mechanistic differences in the process of gating, but also to differences in the biosynthesis of the different families. For example, *KCNA* channels depend on a N-terminal T domain for tetramerization, while KCNH channels rely most primarily on C-terminal tetramerization domains, although there is indication that N-terminal domains affecting multimerization also exist^54^. Perhaps this results in inactive N-terminal tetramers of *KCNA* channels, while the correct association of KCNH channels would be favoured by their multimerization domains. However, *KCNQ* channels also have C-terminal tetramerization domains^55^,^56^ as KCNH, and we did not observe functional channels in this family either.

Finally, our data open new unanswered questions. Thus, the specific step(s) of the synthesis, processing, assembly, quality control or trafficking pathways at which the modules come together remain to be established. Also, the molecular determinants for specificity of recognition of the two channel halves are unknown, although our preliminary experiments suggest that at least the N terminus can be important to build fully functional K_V_11.1 channels. Further work will be necessary to provide adequate answers to these questions.

## Methods

### Molecular biology

The initial split channels were generated from K_V_10.1 or K_V_10.2 in the pSGEM oocyte expression vector (M. Hollmann, Bochum^57^) by inserting at the chosen position in a ‘StopXStart’ cassette, where *X* is a particular restriction enzyme flanked by the TGA stop and ATG start codons. Sequences of oligonucleotides used are listed in Supplementary Table 1. In the case of the N-terminal demi-channel, *X* was a previously absent restriction enzyme. The fragment was then excised with *X* and a second single cutter from the polylinker and ligated into the empty pSGEM. For the C-terminal demi-channels, *X* was chosen among already present single cutters, now becoming a double cutter. Restriction with this enzyme and subsequent religation of the larger DNA fragment resulted in the deletion of the N-terminal part of the sequence. K_V_10.1 was split by inserting a ‘StopBamHIStart’ or ‘StopEag1Start’ cassette after position Y347. For K_V_10.2, ‘StopHindIIIStart’ or ‘StopEag1Start’ cassettes were used after Y344. Subsequent split channels were generated by deleting the sequence after the start codon up to the desired position (C-terminal constructs) or the sequence between the desired position and the stop codon (N-terminal constructs).

The split channels for K_V_11.1 were generated as PCR fragments containing the desired coding sequences that were inserted into the pSP64A+ vector as HindIII–BamHI fragments. The N-terminal fragment was synthesized using a sense oligonucleotide containing a HindIII site, a Kozak’s signal and the sequences for the initial eight K_V_11.1 residues together with an antisense oligonucleotide carrying the coding sequence for residues 535 to 545, a stop codon and the BamHI-recognition site. For the C-terminal K_V_11.1 fragment synthesis, the sense oligonucleotide was designed to contain a HindIII site, a Kozak’s sequence and the start codon followed by the 546 to 557 K_V_11.1 coding sequence, whereas the antisense oligonucleotide covered the last K_V_11.1 residues (1,049–1,059) and carried a stop codon and the BamHI-recognition sequence. The S620T C-terminal fragment was generated the same way using a template carrying the mutation^41^. Sequences of oligonucleotides used are listed in Supplementary Table 1.

Deletions and point mutations were generated using the Quick Change Kit (Agilent Technologies).

Tagged fragments were generated by overlapping PCR. Sequences of oligonucleotides used are listed in Supplementary Table 1. The 5-Myc tag was obtained by PCR from a tagged K_V_7.2 construct (a generous gift from A. Villarroel, Biophysics Unit, UPV-EHU^58^) and overlapped to the K_V_10.1 N-terminal demichannel. The 4-HA tag was generated by insertion of a synthetic oligonucleotide encoding two HA tags into a fragment with two additional HA tags generated by PCR and overlapped to the C-terminal constructs.

All constructs were confirmed by sequencing.

### cRNA synthesis and oocyte microinjection

cRNAs from pSGEM and pSP64A+ constructs were prepared *in vitro* from linearized templates using the T7 or SP6 promoters, respectively, and the mMessage mMachine kit (Ambion). *Xenopus laevis* oocytes were microinjected with a total amount of cRNA ranging from 5 to 25 ng using similar quantities of split channels cRNAs in a final volume of 50 nl. Oocytes were kept at 18 °C in ND96 (96 mM NaCl, 2 mM KCl, 0.2 mM CaCl_2_, 2 mM MgCl_2_, 0.5 mM theophylline, 5 mM HEPES, pH 7.5) or OR-2 (82.5 mM NaCl, 2 mM KCl, 2 mM CaCl_2_, 2 mM MgCl_2_, 1 mM Na_2_HPO_4_, 10 mM HEPES, at pH 7.5) solutions.

### Surface biotinylation

We used the Surface Biotinylation kit (Pierce). Oocytes expressing the corresponding constructs were washed with PBS and incubated for 30 min at 4 °C with 1 mg ml^−1^ sulfo-NHS-biotin under continuous agitation. The reaction was stopped by addition of 25 μl quencher. Oocytes were then homogenized by pipetting through 1,000 and 100 μl tips in 20 μl per oocyte 1% Triton-X-100, 150 mM NaCl, 20 mM Tris-HCl, 5 mM MgCl_2_, 5 mM EDTA and protease inhibitors (Complete; Roche). The homogenate was incubated 30 min on ice and centrifuged twice at 20,000*g* for 3 min. The pellet was discarded and the clarified extract was incubated with Neutravidin resin for 60 min at room temperature. The resin was washed three times and finally the bound proteins were eluted by incubation with NuPage LDS sample buffer for 60 min at room temperature. The eluted proteins were concentrated by precipitation with trichloroacetic acid, and then reconstituted in LDS sample buffer and separated by polyacrylamide gel electrophoresis.

### Immunoprecipitation and immunobloting

Ninety-six hours after injection of cRNA coding for the N-terminal K_V_10.1 demi-channel labelled with 5 × Myc plus the C-terminal 4 × HA-tagged demi-channel, 30 oocytes per group were collected and lysed as above. The pellet was discarded and the supernatant was precleared 1 h at 4 °C with 25 μl Protein G-Magnetic Beads (New England Biolabs). Immunoprecipitation was performed for 1 h at 4 °C on the precleared lysate with 3 μg anti-c-Myc (Santa Cruz cat # sc-40), 2 μg anti-HA (Roche cat. #11867423001) or 2 μg control mouse IgG 2b (Abcam) and 90 min with 50 μl Protein G-Magnetic Beads. The beads were washed three times with 0.1% Triton-X-100, 50 mM Tris-HCl, 300 mM NaCl, 5 mM EDTA plus protease inhibitors. Immunoprecipitated proteins were recovered by heating at 70 °C for 10 min in LDS sample buffer (Invitrogen). Samples to be deglycosylated with PNGase F (Sigma) were resuspended in 16.5 μl water to be digested overnight at 37 °C. For immunoblot analysis, total protein was separated by SDS–polyacrylamide gel electrophoresis, probed with anti-HA (1:1,000, Roche cat. #11867423001) or anti-c-Myc (1:1,000, Sigma cat #M4439). The membranes were stripped and reprobed against the epitope used to immunoprecipitate. Secondary antibodies (HA conjugated) were used at 1: 10,000 dilution (mouse, GE Healthcare #NA931V, rabbit, GE Healthcare #NA934V and rat (Jackson Immunoresearch #112-035-006). Crude extract from 1/2 oocyte was used as input control.

For Blue native electrophoresis, oocytes were washed and homogenized in 0.1 M sodium phosphate buffer, pH 8.0 containing: 0.4 mM Pefabloc SC (Fluka, Buchs, Switzerland) plus 1.5% digitonin (Fluka). Extracts were incubated 10 min on ice and cleared at 25,000*g* for 10 min. 5 μl extract (1/2 oocyte) were mixed with 20 μl Native Sample Buffer plus 0.375% G250 (NuPAGE) and run in 4–16% precast Native Gels (Invitrogen) at 150 V in dark blue buffer for 45 min and at 250 V in light blue buffer. Semi-dry blotting was performed on polyvinylidene difluoride membranes, and immunodetection was performed as above. The anti-K_V_10.1 antibody (polyclonal 9391 (ref. 59)) was used at a dilution of 1:1,500.

Uncropped images of blots are presented in Supplementary Fig. 3.

### Electrophysiology

Two-electrode voltage-clamp recordings were performed at room temperature 2–5 days after injection, using Turbo TEC-10CD and -10C amplifiers (NPI electronics). The intracellular electrodes had resistances of 0.5–1.0 MΩ when filled with 2 or 3 M KCl. For K_V_10 recordings, the extracellular measuring solution contained 115 mM NaCl, 2.5 mM KCl, 1.8 mM CaCl_2_, 10 mM HEPES/NaOH, pH 7.2, with or without the indicated concentrations of MgCl_2_. K_V_11.1 currents and those of the K_V_10.1/K_V_11.1 mixed demi-channels were routinely recorded in OR-2 medium. For recordings in high extracellular K^+^, KCl was increased to 50 or 60 mM substituting NaCl. Oocytes showing membrane potentials more positive than −30 mV and holding currents bigger than 200 nA at −80/−100 mV after impalement with the first and second electrode, respectively, were discarded. Data acquisition and analysis were performed with the Pulse-PulseFit (HEKA Electronics) and IgorPro (WaveMetrics) software packages. Ionic currents sampled at 1 KHz were elicited using the voltage protocols indicated in the graphs. A *P/n* method was used for leak and capacitive current subtraction, except in the case of Cole–Moore protocols, where no subtraction was performed. For K_V_11.1, the voltage dependence of activation was assessed by standard tail current analysis using depolarization pulses of variable amplitude. For very rapidly deactivating constructs, fitting the relaxation of the tail currents and extrapolating the magnitude of the decaying current to the time the depolarizing pulse ended were used to determine the amount of current passing through channels opened on depolarization without influence of rapid inactivation. Tail current magnitudes normalized to maximum were fitted with a Boltzmann function to estimate the *V*_1/2_ and equivalent gating charge (*z*_g_):


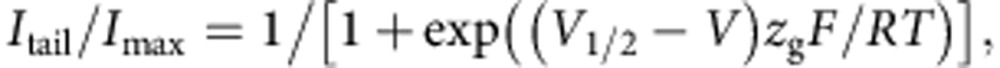


where *V* is the test potential and *F*, *R* and *T* are the Faraday constant, gas constant and absolute temperature, respectively. The time course of voltage-dependent activation was studied using an indirect envelope-of-tail-currents protocol, varying the duration of depolarization prepulses, and following the magnitude of the tail currents on repolarization. The time necessary to reach a half-maximal tail current magnitude was used to compare the speed of activation of the different channels. The rates of deactivation were determined from negative-amplitude biexponential fits to the decaying phase of tail currents using a function of the form:


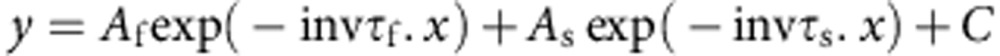


where *τ*_f_ and *τ*_s_ are the time constants of fast and slow components, *A*_f_ and *A*_s_ are the relative amplitudes of these components and *C* is a constant. In this case, the first cursor of the fitting window was advanced to the end of the initial hook because of the recovery of inactivation. Onset of fast inactivation was studied after activation and inactivation of the currents with a prepulse to positive voltages, followed by a second short prepulse to around −100 mV used to recover the channels from inactivation, and a subsequent test pulse to different voltages to reinactivate the channels. Time constants for the onset of inactivation were obtained from current traces fitting a single-exponential function to the decaying portion of the currents during the test pulses.

## Author contributions

E.L., J.C.G.-P., P.d.l.P. and A.P.T. generated the constructs. E.L., J.C.G.-P., A.P.T., J.F.-T., U.L., F.B. and L.A.P. collected the data. E.L., J.C.G.-P., P.d.l.P., W.S., F.B. and L.A.P. designed the study. F.B., P.d.l.P. and L.A.P. wrote the manuscript, all authors discussed the results and commented on the manuscript.

## Additional information

**How to cite this article:** Lörinczi, É. *et al*. Voltage-dependent gating of KCNH potassium channels lacking a covalent link between voltage-sensing and pore domains. *Nat. Commun.* 6:6672 doi: 10.1038/ncomms7672 (2015).

## Supplementary Material

Supplementary Information Supplementary Figures 1-3 and Supplementary Table 1
