# Supplementary material for: Voltage-dependent gating of KCNH potassium channels lacking a covalent link between voltage-sensing and pore domains
Source: Nat Commun. 2015 Mar 30;6:6672. doi: 10.1038/ncomms7672 (PMC4389246; doi:10.1038/ncomms7672)
Supplement: Supplementary Information — Supplementary Figures 1-3 and Supplementary Table 1 [file ncomms7672-s1.pdf]

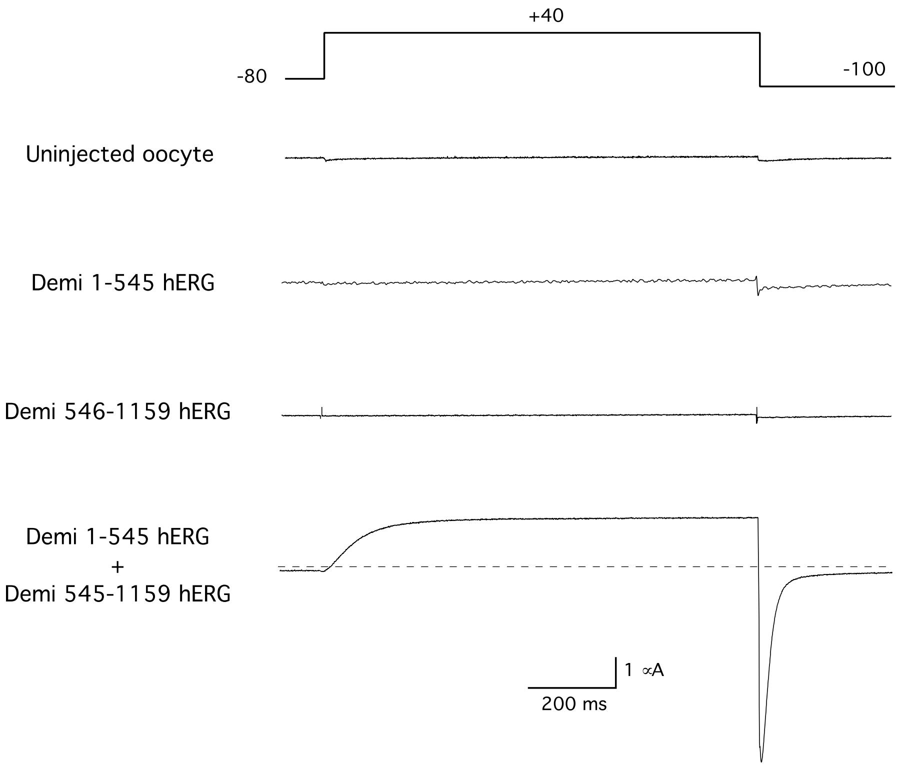

Supplementary Figure 1. Absence of functional expression upon injection of separate N- and C-terminal HERG demi-channels. Representative currents obtained in 50 mM extracellular K^+ with the protocol shown at the top from uninjected oocytes (*top)* and from cells injected with cRNA encoding the N- (*Demi 1-145 hERG)* and C-terminal (*Demi 546-1159 hERG)* halves of HERG. A current trace from an oocyte expressing Y545 Split HERG is shown for comparison at the *bottom*.

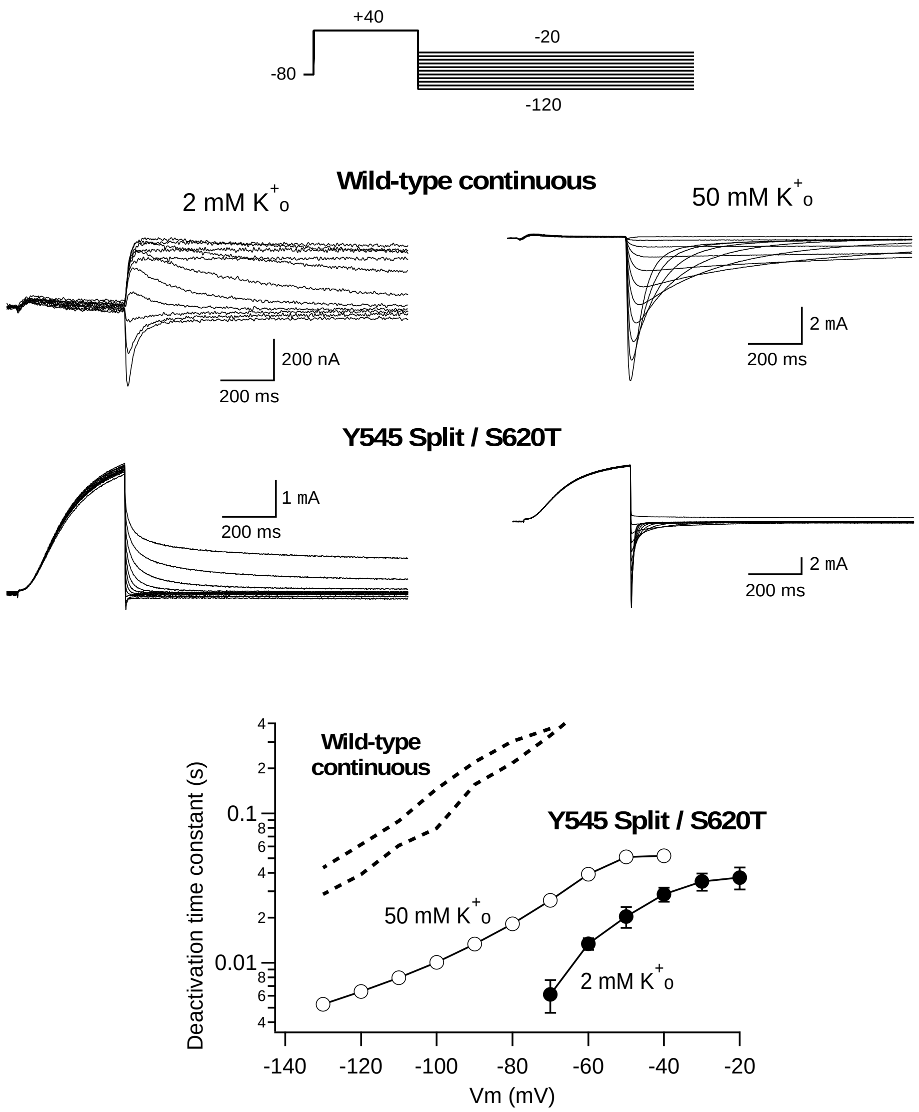

Supplementary Figure 2. Comparison of deactivation properties in wild-type continuous and and Y545 Split HERG channels carrying a S620T point mutation in the pore module that minimizes inactivation. Representative families of current traces obtained with the protocol shown are illustrated at the *top*. Extracellular solutions containing 2 (*left*) and 50 mM (*right*) K^+ were used as indicated. Plots of fast deactivation time constant values at different repolarisation voltages for splits in 2 (*closed circles*, n=4) and 50 mM external K^+ (*open circles*, n=4), and for wild-type non-split channels (*black* and *grey dashed lines*, respectively), are shown at the *bottom*.

Figure 2a: 1. Marker; 2. Continuous (total). 3 Continuous (biotinylated,30µL) 4. Split channel, biotinylated. 5. C Terminus, biotinylated. 6. Control; 7 Marker. The Figure shows only the lanes corresponding to pulled-down, biotinylated samples.

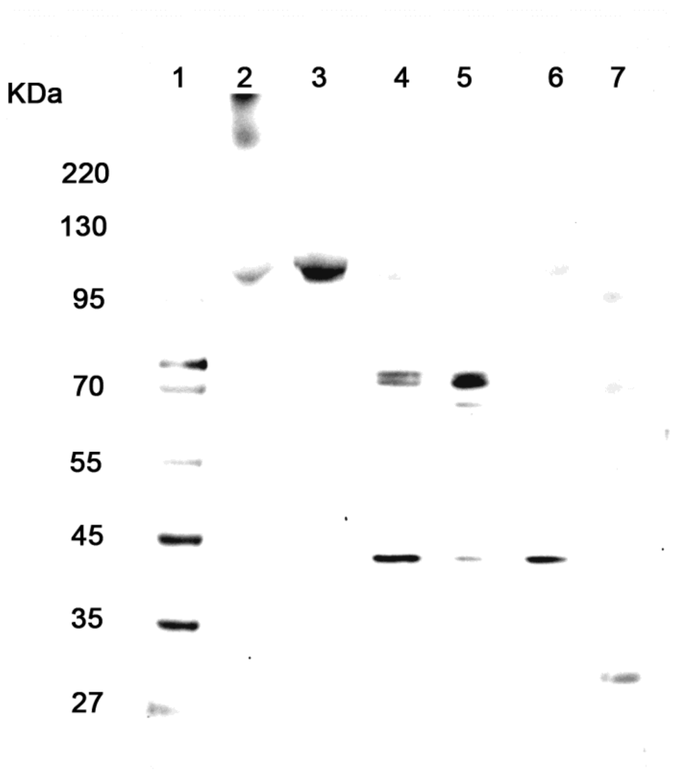

Figure 2b

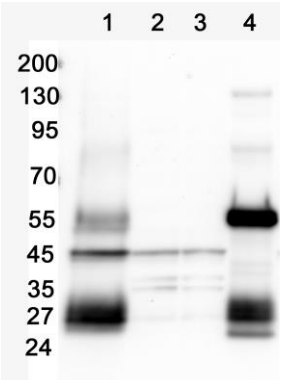

Figure 2c

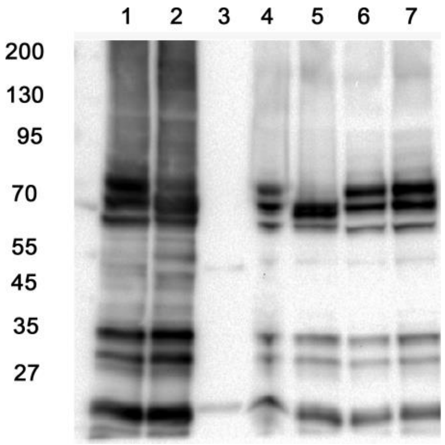

In the Figure, lanes 5-7 (eluates from IP, unbound protein) were omitted for clarity.

Figure 2d

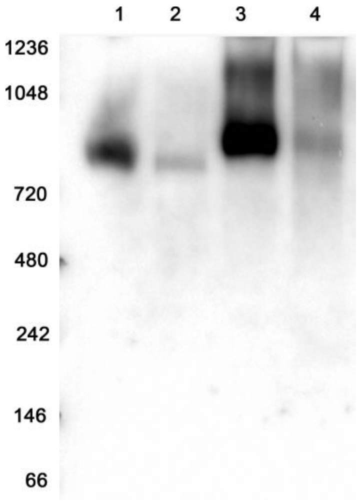

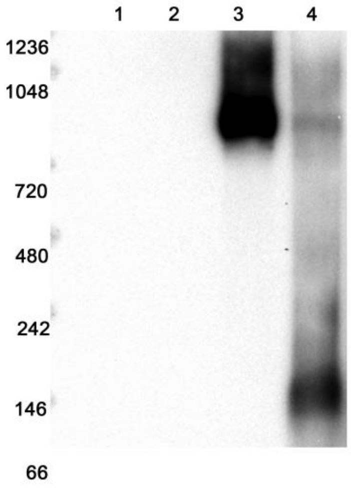

In the Figure, lane 1 (continuous $K_V10.1$, 100 ng injection) was omitted for clarity.

Supplementary Figure 3. Images of uncropped blots

Supplementary Table I. Sequences of oligonucleotides

Channel	Purpose	Sequence (5'-3')
K_V10.1	Insertion of stop codon, *KpnI* site and start codon	ccactacattgaatattgaggtaccatgggagctgctgtgctggtcc
K_V10.1	Insertion of Stop codon, *Eag1* site and start codon	ccactacattgaatattgacggccgatgggagctgctgtgctggtcc
K_V10.1	Insertion of stop codon, *BamHI* site and start codon	ccactacattgaatattgaggatccatgggagctgctgtgctggtcc
K_V10.1	Deletion of residues from L341 of N-terminal demichannel	cgagtggcccgtaagctgtgagaggtctatttaaaa
K_V10.1	Deletion of residues up to A350 of C-terminal demichannel	tggcggccgatggctgtgctggtc
K_V10.1	5MycTagging, 1st template and overlapping PCR, forward	ggaccccgagctgctgggaggaaagctatggagcaaaagctc
K_V10.1	5MycTagging, 1st and 2nd template PCR, reverse	cggccggaattccgggcccgccggaccccgagctgctgggagg
K_V10.1	5MycTagging, 2nd template PCR, forward	cctgcccccagccatggtcatgtcgcccaagctctccatttc
K_V10.1	5MycTagging, 3rd template PCR, forward	gaaatggagagcttgggcgacatgaccatggctgggggcagg
K_V10.1	5MycTagging, 3rd template and overlapping PCR, reverse	gaatcatcctcaattggctgtttgaaagct
K_V10.1	HA tagging, 1st HA forward	caactagttgtacagatcaagcgggatgccctgc
K_V10.1	HA tagging, 1st HA reverse	cgcataatctgggacgtcataagggtagctggctccaaaaatgtctct
K_V10.1	HA tagging, 2nd HA forward	caactagttgtacagatcaagcgggatgccctgc
K_V10.1	HA tagging, 2nd HA forward	atggatccctctagattacgcataatccggaacgtcataagggtatcccg cataatctgggacgtcataagg
K_V10.1	Synthetic HA tags 3 and 4, sense	ccggtacgtcataagggtatcccgcataatctggaacgtcataagggtat cccgcataat
K_V10.1	Synthetic HA tags 3 and 4, antisense	ccggattatgcgggatacccttatgacgttccagattatgcgggataccct tatgacgta
K_V10.1	Subcloning 4HA into pSGEM, fragment 1 and overlapping PCR, forward	aaggaggacatcaaggccttaaacgccaaa
K_V10.1	Subcloning 4HA into pSGEM, fragment 1reverse	ttttaaatagacctctcacgcataatccgg
K_V10.1	Subcloning 4HA into pSGEM, fragment 2, forward	ccggattatgcgtgagaggtctatttaaaa
K_V10.1	Subcloning 4HA into pSGEM, fragment 2 and overlapping PCR, reverse	ccctcgaggtcgacggtatcgataagcttg
K_V10.1	Mutant G440S	caccagtgtgggcttttcgaacatcgccccatc
K_V10.2	Insertion of stop codon, *Eag1* site and start codon	ccattacctagaatattgacggccgatgggagcagcagtcctcgtgctcc
K_V10.2	Insertion of stop codon, *HindIII* site and start codon	ccattacctagaatattgaaagcttatgggagcagcagtcctcgtgctcc
K_V10.1	Mutant R336Q	tccgtcttgggcaagtggcgcgcaagctggaccac
K_V11.1	Mutant G628S	ccagtgtgggcttcagcaacgtctctccc
K_V11.1	Synthesis N-terminal demi-channel, forward	ggaagctttcaggatgccggtgcggaggggccacgtc
K_V11.1	Synthesis N-terminal demi-channel, reverse	ggggatcctagtactctgagtagcgatccagcttccgc
K_V11.1	Synthesis C-terminal demi-channel, forward	ggaagctttcaggatgggcgcggccgtgctgttcttgctcatgtgcacc
K_V11.1	Synthesis C-terminal demi-channel, reverse	ggggatcctaactgcccgggtccgagccgtgtctgtgcagggg
